# Prevalence and risk factors associated with high-risk human papillomavirus infection among women living with HIV (WLWH) at a tertiary health facility in Accra, Ghana

**DOI:** 10.1371/journal.pone.0303535

**Published:** 2024-05-30

**Authors:** Frederick Agyare Gyane, Emefa Modey, Ernest Maya, Evelyn Yayra Bonney, Araba Abaidoo-Myles, Elijah Paintsil, Kwasi Torpey

**Affiliations:** 1 School of Public Health, University of Ghana, Accra, Ghana; 2 Noguchi Memorial Institute for Medical Research, Accra, Ghana; 3 Yale School of Medicine, New Haven, CT, United States of America; University Federico II of Naples, ITALY

## Abstract

**Background:**

Women living with HIV (WLWH) have high risk of developing cervical cancer. High- risk Human papillomavirus (hrHPV) is the single most important cause of cervical cancer. Vaccination for and early detection of pre-malignant cervical changes, through cervical cancer screening contributes to prevention of cervical cancer. This study sought to determine the prevalence of HPV among WLWH, genotypes present and the risk factors associated with cervical cancer development.

**Methods and findings:**

An analytical cross-sectional study of 250 sexually active women aged 18 years and above, attending HIV clinic at a tertiary health facility in Accra. Demographic data collection and risk factor assessments were done using interviewer-administered questionnaire, and patient records. Cervical swabs were collected and tested for HPV using real-time PCR assays. Genotype analysis was performed on 92 samples. Descriptive statistics and logistic regression analysis were used to establish associations between hrHPV and risk factors among WLWH. Approximately 60% of study participants tested positive for HPV. The prevalence of hr-HPV among WLH was 44.4%. Factors identified to be protective of hrHPV were employment (AOR = 0.19, 95% CI = 0.06, 0.56, p = 0.003) and highly active antiretroviral therapy (HAART) Tenofovir-Lamivudine-Ritonavir-Lopinavir (TLRL) (AOR = 0.30, 95% CI = 0.09, 0.95, p = 0.04). Women with HIV diagnosis within 6 to10 years (AOR = 4.89, 95% CI = 1.05, 22.70, p = 0.043) and diagnosis >10 years (AOR = 8.25, 95% CI = 1.24, 54.84, p = 0.029) had higher odds of hrHPV. Approximately 25% of samples analysed tested positive for hr-HPV group 1 (genotypes 16, 18, 31, 33, 35, 39, 45,51, 52, 56, 58, 69) and 46.8% for multiple HPV genotypes.

**Conclusion:**

A high prevalence of genotypes that include high risk genotypes 16 and 18 and multiple HPV infections was found among WLWH. Almost half of the women screened had high-risk HPV and were prone to cervical cancer without their knowledge. Regular HPV screening is recommended for high-risk patient groups.

## Background

Human papilloma virus (HPV) infection is the commonest sexually transmitted infection globally [1]. Cervical cancer is the most common HPV-related disease [2, 3]. The alpha genus of HPV that causes pathological conditions in humans is subclassified into low-risk and high-risk subtypes based on cancer causing ability. Low risk Human papilloma virus (lrHPV) subtypes cause condyloma acuminata (genital warts) but the high-risk Human papilloma virus (hrHPV) subtypes cause cancer [4]. An estimated 99.7% of cervical cancer cases are caused by hrHPV infection [3]. The emergence of HIV has contributed to changes in the clinical pattern of STIs [5]. There is evidence to suggest that the presence of HIV infection in women increases the risk of developing cervical carcinoma by twenty-fold [6]. Cervical carcinoma is the fourth commonest cancer in women worldwide and the leading cause of cancer deaths in women in Ghana [7, 8]. Women living with HIV (WLWH) have a high risk of developing cervical cancer [9, 10].

Screening methods for cervical cancer are the same for all women regardless of HIV status and the decision on what test to use may be informed by resources available [11]. The screening methods are classified into 3 categories: visual based methods such as Visual Inspection with Acetic acid (VIA) and Visual Inspection with Lugol’s Iodine (VILI); cytology-based methods such as the conventional Papanicolaou (Pap) smear and Liquid Based Cytology (LBC) testing; and HPV deoxyribonucleic (DNA) testing [12–15]. The unique advantages of VIA lie in the ability to train lower cadre staff to perform the test and the option of screening and treating concurrently [11]. However, the high false positive rate in the utilisation of VIA remains a disadvantage.

Several risk factors of HPV infection have been reported in different study populations. These included the age at coitarche, number of sexual partners, and sexual behaviour of partners. The cervix is affected by other factors that make it more susceptible to infectivity with HPV. These include the presence of infection with other sexual transmitted diseases (STIs), cigarette smoking, pregnancy, poor use of barrier methods, prolonged use of hormonal contraception, immunosuppression including HIV infection, and non-adherence to antiretroviral therapy (ART) in WLWH. The presence of HIV infection is notably the biggest risk factor for cervical cancer [9, 10, 16].

The prevalence of HIV in the adult population in Ghana is 1.7%. However, some regions and districts such as the greater Accra Region have higher prevalence of 2.47% [17, 18]. Although studies on HPV among HIV women in Ghana are limited, the few reported studies show higher prevalence of HPV among HIV women [19, 20]. Despite having a national policy on cervical cancer screening for premalignant cervical lesions, there is no nationwide program to implement the policy [21]. Additionally, following the introduction of the HPV vaccine in Ghana in 2013 through support from the Global Alliance for Vaccines and Immunisations (GAVI), vaccination remains low. Poor access, lack of knowledge and cost have been reported as barriers to uptake [22]. The estimated population of all women that have been screened by pap smear is low at 2.4%, most of which has been opportunistic [23, 24].

Screening and vaccination remain critical to efforts aimed at cervical cancer elimination. The distribution of HPV genotypes and the causal role of different genotypes, show variation depending on the geographical region. The International Agency for Research on Cancer (IARC) classifies HPV genotypes based on carcinogenicity. Genotypes classified as carcinogenic (hr-HPV) are (Group 1: HPVs 16, 18, 31, 33, 35, 39, 45, 51, 52, 56, 58, 59). Possible hr-HPVs that are possibly carcinogenic include HPVs 26, 53, 66, 67, 69, 70, 73 and 82 are Group 2B. Then HPVs 6, 11, 40, 42, 43, 44, 55, 61, 81, 83 are classified as the low-risk HPV (lr-HPV) [25]. Recent evidence from Ghana HPV 45 and 59 as the commonest hr-HPV found in samples in Ghana [26].

Understanding the sociodemographic and clinical factors associated with HPV will provide insight into the needed input and strategies required to reduce the prevalence of cervical cancer among all women with particular emphasis on WLWH that are most at risk. This study sought to generate evidence of the prevalence and identify risk factors associated with high-risk HPV infection among WLWH to inform strategies and care of WLWH in Accra.

## Material and methods

### Study design and population

This was an analytical cross-sectional study implemented in a tertiary hospital in Accra, Ghana. The study population consisted of women aged 18 years and above who were sexually active. Participants were conveniently sampled from the HIV clinic at the Hospital. Women were approached individually and provided with information on the study. Ethical approval was granted from the hospital authorities with approval number 37MH-IRB/MAS/IPN/544/2.1. Written informed consent was sought from all participants prior to enrolment into the study. Interviews were conducted, and samples were collected for analysis after consent was obtained.

### Data collection

Participant recruitment occurred from September 25, 2021 and ended on January 7, 2022. A questionnaire was developed to collect baseline data on sociodemographic, risk factors for HPV infection (sexual partners, pregnancy history, smoking history, contraceptive use, other sexually transmitted infections, and use of immunosuppressive drugs) and previous cervical screening and treatment. Cervical swabs were also taken from participants and tested for HPV and its genotypes using real-time PCR assays. Additionally, data was extracted from hospital records to access information on the care received by participants.

A qualified public health nurse from the Public Health Division of the facility was retrained to perform pelvic examinations and collect cervical samples during medical visits at the HIV clinic from Monday to Friday over the data collection period. This involved the inspection of the external genitalia for genital warts, syphilitic chancres, polyps, varices, herpes vesicles, and any other lesions. A non-lubricated disposable bivalve speculum was inserted to visualize the vaginal walls and cervix. Using an Ayres spatula and a cytobrush, cervical exfoliated cells were collected by rotating the spatula and brush gently in a clockwise direction 360 degrees, three (3) times. The specimen collected was smeared evenly on a glass slide and fixed with 95% alcohol. The slide was then air dried.

### PCR amplification and analysis

The cervical scrapings from the cytobrush were transported in cool boxes to the Department of Virology of the Noguchi Memorial Institute for Medical Research (NMIMR), University of Ghana. The specimens were stored at -20 degrees Celsius until HPV DNA extraction. Specimens were thawed and 1.2 ml of Phosphate—Buffered Saline (PBS) transport medium added into the tube and then vortex-mixed the sample for 15 min. After vortex-mixing, the swab was removed from the tube. HPV DNA was extracted from specimens using the QIAamp DNA extraction kit from QIAGEN following the manufacturer’s instructions.

The DNA extracts were subjected to real time PCR using the Mole Biosciences HPV Nucleic Acid Amplification Test Kit, that detects genotypes of HPV. For, each sample, a total volume of 25μl, made up 10 μl reaction buffer, 9.3 μl of primer/probe mix, 0.7 μl of enzyme and 5 μl of template DNA was prepared and run in ABI 7500 real time PCR system. Thermal conditions were 50°C for 5minutes, followed by 95°C for 2mins then 5cycles of 94°C for 15 seconds and 54°C for 1minute followed by 40 cycles of 94°C for 15 seconds and 52°C for 1minute. The amplification products were analysed based on the presence or absence of specific reporter dyes. An additional fluorescent probe CY5 was used as internal control. Positive and negative controls were included in each set of PCR runs. A cycle threshold (CT) of 36 was used as cut off. Any sample with CT > 36 for a particular genotype was deemed negative for that genotype while samples with CT ≤ 36 were recorded as positive. For the internal control, CT 32 was the cut-off point.

### Data analysis

Descriptive statistics was used to summarize data on the demographic and risk factors. The prevalence of hr-HPV was defined as the proportion of positive results in the total study population, and the 95% confidence intervals calculated. A bivariate logistic regression analysis was performed to identify associations between sociodemographic, sexual behavioural characteristics and the presence of hr-HPV infection using odds ratios and 95% confidence intervals. The level of significance was set at 5%.

## Results

### Demographic characteristics of study participants

The results of the 250 participants show a mean age of 43.8 years (±11.2). Relatively few women (18.4%) had no formal education. The highest level of education attained was tertiary (14.4%) but the majority of women had primary education (41%). An estimated 89% of the study participants were employed although monthly income was less than GHC500 in 34% (Table 1). Only 8% of the women had ever been screened for cervical cancer.

**Table 1:** Background characteristics of women attending HIV clinic at a tertiary hospital in Accra, Ghana.

Characteristics	hrHPV negative	hrHPV positive	Total
	n = 139	n = 111	N = 250
**Age groups**			
<30 years	14(12.6)	12(10.8)	26(10.4)
30–39 years	34(30.6)	32(28.8)	66(26.4)
40–49 years	46(41.4)	38(34.2)	84(33.6)
50–59 years	28(25.2)	23(20.7)	51(20.4)
60+years	17(15.3)	6(5.4)	23(9.2)
**Level of education**			
No formal education	25(22.5)	21(18.9)	46(18.4)
Primary	58(52.2)	45(40.5)	103(41.2)
Secondary	36(32.4)	29(26.1)	65(26.0)
Tertiary	20(18.0)	16(14.4)	3614.4)
**Employment status**			
Unemployed	11(7.9)	15(13.5)	26(10.4)
Employed	128(92.1)	96(86.5)	224(89.6)
**Marital status**			
Single	32(28.8)	30(27)	62(24.8)
Married	54(48.6)	38(34.2)	92(36.8)
Divorced/Separated	22(19.8)	27(24.3)	49(19.6)
Widowed	31(27.9)	16(14.4)	47(18.8)
**Monthly income**			
Not indicated	29(26.1)	35(31.5)	64(25.6)
<GH₵500	58(52.2)	28(25.2)	86(34.40
GH₵500–1000	36(32.4)	43(38.7)	79(31.6)
GH₵1001–2000	8(7.2)	1(0.9)	9(3.6)
GH₵2000+	8(7.2)	4(3.6)	12(4.8)
** Ever smoked**			
No	132 (55.0)	108 (45.0)	240(96.0)
Yes	7 (70.0)	3 (30.0)	10(0.4)
TOTAL	139 (55.6)	111(44.4)	250(100.0)

### Prevalence of HPV and hrHPV among study participants

The results of the HPV test and cervical cytology carried out as part of the study showed that 150 (60%) of the 250 women attending HIV clinic tested positive for HPV. Further testing showed that out of all the participants, 111(44)% were infected with hrHPV.

### HPV genotyping by Real -time PCR

Due to the non-availability of the test kit at the time of this study, testing was done on only the first 92 samples taken. A total of 92 samples were amplified to detect the genotype of HPV present. Out of the 92 samples tested, 66 (71.7%) tested positive for at least one HPV group and the remaining tested negative for all 3 groups. Majority of the samples (25%) tested positive for Group 1 genotypes that included high risk genotypes 16 and 18 Dual infections of lrHPV and group 1 were observed in 20.7% of the study population. The lowest prevalence occurred in lrHPV types (8.7%) and 3 (7.6%); dual infections between Group 1 and lrHPV and that between Groups 2B and lrHPV were 1.1% and 2.2% respectively. Six samples (6.5%) tested positive for all three groups (Table 2).

**Table 2:** HPV genotyping results for 92 samples.

Genotypes	No. of Positive samples	Percent (%)	
Group 1	23	25.0	
Group 2B	7	7.6	
lr-HPV & Group1	19	20.7	
lr-HPV & Group 2B	1	1.1	
Group 1 & 2B	2	2.2	
lr-HPV	8	8.7	
Positive for all groups	6	6.5	
Negative for all groups	26	28.3	
**Total**	**92**	**100.0**	
Group 1	16, 18, 31, 33, 35, 39, 45,51, 52, 56, 58, 69		
Group 2B	26, 53, 66, 73, 82		
lr-HPV	6,11,42,43,44, 81		

### Sexual and reproductive characteristics/Cervical cancer screening

The results of the HPV test and cervical cytology carried out as part of the study is as shown in Table 3. Among women with hr-HPV, approximately 36% of those who had ever used contraception were using a method of contraception at the time of the study. Of the study participants that had ever had an STI, gonorrhoea was the commonest STI reported (Table 3).

**Table 3:** Sexual and Reproductive characteristics of women attending HIV clinic at a tertiary hospital in Accra.

Characteristics	Non hrHPV	hrHPV	Total
	n = 139	n = 111	N = 250
**Age at first sex**			
Can’t remember	28 (20.1)	15 (13.5)	43(17.2)
<15 years	2 (1.4)	1 (0.9)	3(1.2)
15–19 years	57 (41.0)	58 (52.3)	115(46.0)
20+ years	52 (37.4)	37 (33.3)	89(35.6)
**Ever been screened for cervical cancer**			
No	128(92.1)	102(91.9)	230(92.0)
Yes	11(7.9)	9(8.1)	20(8.0)
**Ever been pregnant**			
No	8 (5.7)	7 (46.7)	15(6.0)
Yes	131 (94.2)	104 (44.3)	235(94.0)
**Number of pregnancies (n = 235)**			
One	17(12.2)	18(16.2)	35(14.0)
2–3	42(30.2)	33(29.7)	75(30.0)
4- - 5	48(34.5)	32(28.8)	80(32.0)
6+	24(17.3)	21(18.9)	45(18.0)
**Type of sexual activity**			
Vaginal	136 (97.8)	108 (97.3)	244(97.6)
Non-vaginal	3 (2.2)	3 (2.7)	6(2.4)
**Number of sex partners**			
1	22 (15.8)	19 (13.7)	69(27.6)
2 to 3	67 (48.2)	47 (42.3)	72(28.8)
4+	28 (20.1)	35 (31.5)	8032.0)
Undisclosed	22 (15.8)	10 (9.0)	11(4.4)
**Ever used contraception**			
No	80 (57.6)	64 (57.7)	144(57.6)
Yes	59 (42.4)	47 (42.3)	106(42.4)
**Current use of contraception (n = 106)**			
No	41(69.5)	30(63.8)	71(28.40
Yes	18(30.5)	17(36.2)	35(14.0)
**Ever had STI**			
No	125(89.9)	105(94.5)	230(92.0)
Yes	14(10.1)	6(5.4)	20(8.0)
**Type of STI (n = 20)**			
Can’t remember	3(21.4)	1(16.7)	4(20.0)
Gonorrhoea	11(78.6)	5(83.3)	16(80.0)
**HAART**			
TLD	115 (82.7)	102(91.9)	217(86.8)
ALD	7 (5.0)	0	7(2.8)
TLE	2 (1.4)	3 (2.7)	5(2.0)
TLRL	15 (10.8)	6 (5.4)	21(8.4)
**Duration of HIV Diagnosis**			
<1 year	12 (8.6)	4 (3.6)	16(6.4)
1–5 years	103 (74.1)	82 (73.9)	185(74.0)
6–10 years	18 (12.9)	17 (15.3)	25(10.0)
>10 years	6 (4.3)	8 (7.2)	14(5.6)
** TOTAL**	139 (56.0)	111(44.0)	250

### Risk factors associated with hrHPV among study participants

Multiple factors were found to be associated with hr-HPV. First, WLWH who were employed were less likely to have hr-HPV infection compared with those unemployed (AOR = 0.19, 95% CI = 0.06, 0.56, p = 0.003). For those on ART, TLRL combinations were less likely to have developed hr-HPV (AOR = 0.30, 95% CI = 0.09, 0.95, p = 0.04) when compared with those on TLD combinations (Table 4). However, women earning a monthly income of 500–1000 had 3.71 times the odds of hr-HPV (AOR = 3.71, 95% CI = 1.63, 8.48, p = 0.002) compared to women with less than GHC500 monthly income. Divorced/Separated women also had increased odds of hr-HPV (AOR = 3.14, 95% CI = 1.08, 9.18, p = 0.036) when compared with single women. Furthermore, the duration of HIV diagnosis showed that WLWH who have lived with HIV for 6–10 years had 4.89 times the odds of hr-HPV (AOR = 4.89, 95% CI = 1.05, 22.70, p = 0.043). Women diagnosed with HIV >10 years ago also had 8.25 times the odds of hr-HPV compared with those diagnosed <1yr (AOR = 8.25, 95% CI = 1.24, 54.84, p = 0.029) (Table 4).

**Table 4:** Factors influencing high-risk HPV infection among HIV positive women attending HIV clinic at a tertiary hospital in Accra, Ghana.

Dependent Variable: hr-HPV	AOR	95% CI		P value	AOR	95% CI		P value
		Lower	Upper			Lower	Upper	
**Age**	0.99	0.97	1.01	0.287	0.99	0.96	1.03	0.641
**Educational level**								
No formal education	*Ref*				*Ref*			
Primary	0.92	0.46	1.86	0.824	1.44	0.59	3.49	0.42
Secondary	0.96	0.45	2.05	0.914	1.14	0.44	2.92	0.792
Tertiary	0.95	0.4	2.29	0.913	2.30	0.57	9.24	0.239
**Employment status**								
Unemployed	*Ref*				*Ref*			
Employed	0.55	0.24	1.25	0.154	0.19	0.06	0.56	**0.003**
**Marital status**								
Single	*Ref*				*Ref*			
Married	0.75	0.39	1.44	0.386	1.37	0.51	3.66	0.528
Divorced/Separated	1.3	0.62	2.78	0.482	3.14	1.08	9.18	**0.036**
Widowed	0.55	0.25	1.2	0.135	1.31	0.40	4.31	0.652
**Monthly income**								
<500	*Ref*				*Ref*			
500–1000	2.47	1.31	4.66	0.005	3.71	1.63	8.48	**0.002**
1001–2000	0.26	0.03	2.18	0.213	0.18	0.01	2.60	0.21
2000+	1.04	0.29	3.73	0.957	0.77	0.14	4.23	0.766
Not indicated	2.5	1.28	4.87	0.007	2.45	1.06	5.63	**0.035**
**New sexual partner in past year**								
No	*Ref*				*Ref*			
Yes	1.3	0.78	2.17	0.317	1.49	0.68	3.28	0.317
**Number of partners in past year**								
None	*Ref*				*Ref*			
One	1.08	0.65	1.82	0.758	0.75	0.33	1.75	0.51
Two	1.33	0.36	4.87	0.671	1.20	0.16	8.79	0.86
**Type of sexual activity**								
Vaginal	*Ref*				*Ref*			
Non-vaginal	1.26	0.25	6.36	0.78	1.03	0.14	7.40	0.973
**Partner has partners**								
No	*Ref*				*Ref*			
Unknown	1.34	0.78	2.28	0.285	1.05	0.52	2.09	0.895
Yes	1.59	0.69	3.68	0.274	1.23	0.44	3.42	0.697
**Age at first sex**								
<15 years	*Ref*				*Ref*			
15–19 years	0.93	0.08	11.16	0.957	5.68	0.39	83.46	0.205
≥20 years	1.9	0.92	3.93	0.083	2.69	0.18	39.57	0.47
Can’t remember	1.32	0.62	2.83	0.462	2.04	0.13	31.51	0.61
**Sexual partners since first sex**								
One	*Ref*				*Ref*			
2 to 3	0.81	0.4	1.67	0.57	0.83	0.35	1.98	0.68
4+	1.45	0.66	3.19	0.359	1.20	0.45	3.22	0.716
Undisclosed	0.53	0.2	1.38	0.193	0.70	0.20	2.42	0.571
**Ever been pregnant**								
No	*Ref*				*Ref*			
Yes	0.91	0.32	2.58	0.855	1.22	0.31	4.81	0.781
**Ever smoked**								
No	*Ref*				*Ref*			
Yes	0.52	0.13	2.07	0.357	0.28	0.05	1.57	0.146
**Ever used contraceptives**								
No	*Ref*				*Ref*			
Yes	0.99	0.6	1.65	0.987	0.84	0.45	1.57	0.574
**Ever had any STIs**								
No	*Ref*				*Ref*			
Yes	0.51	0.19	1.37	0.183	0.34	0.10	1.17	0.087
**Duration of HIV disease**								
<1 year	*Ref*				*Ref*			
1–5 years	2.39	0.74	7.68	0.144	2.86	0.72	11.25	0.134
6–10 years	2.83	0.76	10.52	0.12	4.89	1.05	22.70	**0.043**
>10 years	4	0.85	18.84	0.08	8.25	1.24	54.94	**0.029**
**HAART combinations**								
TLD	*Ref*				*Ref*			
TLE	1.69	0.28	10.32	0.569	1.03	0.13	8.21	0.979
TLRL	0.45	1.69	1.21	0.113	0.30	0.09	0.95	**0.04**
ALD	Omitted				Omitted			

COR = Crude Odd Ratio; CI = Confidence Interval; AOR = Adjusted Odds Ratio; Ref = Reference category, Omitted: Omitted category due to collinearity/ few counts TLD–Tenofovir Lamivudine; Dolutegravir; ALD–Abacavir Lamivudine; Dolutegravir; TLE—Tenofovir Lamivudine Emtricitabine; TLRL—Tenofovir Lamivudine Ritonavir Lopinavir.

## Discussion

In our study, we found a high prevalence of HPV infection among WLWH in Ghana. The observed low prevalence of screening is indicative of the need to introduce comprehensive programmes and interventions that can be sustained by the current health care system. A programmatic effort to make available and accessible varied options for screening to enhance surveillance could improve early detection.

Earlier studies in Ghana determining the prevalence of HPV have shown varied levels ranging from 30% in North Tongu [27] to 35% in Accra and Kumasi [28] among women of reproductive age. However, the hr-HPV prevalence among WLWH in this study was estimated as 44.4%. This finding was lower, compared to the available estimates that report 65% among WLWH in the Central region of Ghana, 77.4% among WLWH in Kumasi [20] and 65.6% among WLWH in Cape Coast [19].

The levels of hr-HPV prevalence in sub-Saharan Africa (SSA) countries have been varied [29]. The prevalence of hr-HPV from the current study is higher than the (19.6%) that found among WLWH in cervical cancer screening centers in Ogun and Lagos states, Nigeria [30] and antiretroviral clinic of the Federal Medical Center (36%), Keffi, Nigeria [31]. In Zimbabwe [32], also reported about 25% hr-HPV prevalence which is also lower than the findings from this study. Some other SSA countries such as Burundi, 45.7% [33], Kenya 47% [34] and 64% [35] have been reported.

The burden of HPV and hrHPV genotypes among WLWH identified contributes to the available studies on genotypes present in Ghana [26, 28, 36, 37]. The presence of more Group 1 genotypes that include high risk genotypes 16 and 18 is similar to what has been observed in subSaharan Africa [20, 29, 38]. The availability of current data on the genotype-specific distribution of HPV and hrHPV among vulnerable groups provides evidence for prioritised vaccine recommendations.

The results also indicate that the women with hr-HPV were fairly an adult population. This finding is similar to evidence from Ghana [23] While advanced age has been indicated as a risk factor for persistent HPV infections [39], other studies have reported a more youthful population [25, 27, 37] where majority of participants were below 30 years. The occurrence of hr-HPV among different age groups especially the youth and working population of the economy has public health and economic implications. The contribution of these infections to loss of man-hours or productivity due to morbidity and mortality can exacerbate the economic burden of a country [40] especially low- and middle-income countries like Ghana. This also deepens the relationship between poverty and HIV [41] with a compounding effect due to its co-infection with hr-HPV.

The prevalence of HPV infection has been shown to vary economically [42, 43]. Among employed women, the low hrHPV observed may be a result of these varying socioeconomic factors that predispose women to increased morbidity [44]. Against the backdrop that the National Health Insurance Scheme (NHIS) does not yet cover screening, health care needs of some vulnerable groups of women may not be fully met. Similar findings have been observed in South Africa [45]. Additionally, co-occurrence of HIV with HPV increases the burden of HPV-related cancers and deepens the loss of social and economic quality of life among people living with HIV [46]. It is important to develop health and socio-economic interventions to support those affected to reduce morbidity and mortality [41].

The ART regimen remained an influencing factor of hr-HPV among HIV positive women. From this study, HIV positive women on Tenofovir-Lamivudine-Ritonavir-Lopinavir (TLRL) combinations were less likely to have hr-HPV infection when compared with those on Tenofovir-Lamivudine-Dolutegravir combinations. The finding of ART as an associated factor to hr-HPV has been reported by [47] who indicated that, the duration of providing ART reduced the prevalence of hr-HPV infection in sub-Saharan Africa. This is also in agreement with [30] where lower prevalence of hr-HPV was found among HIV infected women using ART in Nigeria. Other evidence supporting the influence of ART on cytology have been found in Nigeria [48] and Zimbabwe [32].

The finding that longer duration of diagnosis was associated with increased hrHPV implies the effect of long periods of immunosuppression as STIs may be acquired, of which HPV is inclusive [49]. In the absence of effective ARTs, immunosuppression increases the risks of having hr-HPV and related cancers [47]. According to [50], the early initiation and adherence to ART has the potential to reduce the progression of HPV infection. This is because, effective ART reduces viral loads to suppression levels thereby boosting immune system support against HPV- associated clinical conditions and other opportunistic infections [19, 51]. The unavailability of viral load and CD4 count assessment for patients was a limitation of this assessment.

## Conclusion

The higher prevalence of hr-HPV with an increasing duration of HIV infection from the study implies the need for galvanised efforts to reduce morbidity. Reassuringly however, is the low hrHPV among employed women and protective association of HAART TLRL with hrHPV. There is a need for more epidemiological studies to establish this relationship and provide evidence for appropriate treatment options. The findings of the study nonetheless provide evidence of the need for greater surveillance through regular HPV screening among at risk patients groups by clinicians.

## Supporting information

S1 Checklist *PLOS ONE* clinical studies checklist. (DOCX)

S1 File Prevalence and risk factors of HrHPV among WLWH survey. (XLSX)
